# Arterial compliance probe for cuffless evaluation of carotid pulse pressure

**DOI:** 10.1371/journal.pone.0202480

**Published:** 2018-08-16

**Authors:** Jayaraj Joseph, Nabeel P M, Malay Ilesh Shah, Mohanasankar Sivaprakasam

**Affiliations:** 1 Healthcare Technology Innovation Centre, Indian Institute of Technology Madras, Chennai, Tamil Nadu, India; 2 Department of Electrical Engineering, Indian Institute of Technology Madras, Chennai, Tamil Nadu, India; Kurume University School of Medicine, JAPAN

## Abstract

**Objective:**

Assessment of local arterial properties has become increasingly important in cardiovascular research as well as in clinical domains. Vascular wall stiffness indices are related to local pulse pressure (ΔP) level, mechanical and geometrical characteristics of the arterial vessel. Non-invasive evaluation of local ΔP from the central arteries (aorta and carotid) is not straightforward in a non-specialist clinical setting. In this work, we present a method and system for real-time and beat-by-beat evaluation of local ΔP from superficial arteries—a non-invasive, cuffless and calibration-free technique.

**Methods:**

The proposed technique uses a bi-modal arterial compliance probe which consisted of two identical magnetic plethysmograph (MPG) sensors located at 23 mm distance apart and a single-element ultrasound transducer. Simultaneously measured local pulse wave velocity (PWV) and arterial dimensions were used in a mathematical model for calibration-free evaluation of local ΔP. The proposed approach was initially verified using an arterial flow phantom, with invasive pressure catheter as the reference device. The developed porotype device was validated on 22 normotensive human subjects (age = 24.5 ± 4 years). Two independent measurements of local ΔP from the carotid artery were made during physically relaxed and post-exercise condition.

**Results:**

Phantom-based verification study yielded a correlation coefficient (r) of 0.93 (p < 0.001) for estimated ΔP versus reference brachial ΔP, with a non-significant bias and standard deviation of error equal to 1.11 mmHg and ±1.97 mmHg respectively. The ability of the developed system to acquire high-fidelity waveforms (dual MPG signals and ultrasound echoes from proximal and distal arterial walls) from the carotid artery was demonstrated by the in-vivo validation study. The group average beat-to-beat variation in measured carotid local PWV, arterial diameter parameters—distension and end-diastolic diameter, and local ΔP were 4.2%, 2.6%, 3.3%, and 10.2% respectively in physically relaxed condition. Consistent with the physiological phenomenon, local ΔP measured from the carotid artery of young populations was, on an average, 22 mmHg lower than the reference ΔP obtained from the brachial artery. Like the reference brachial blood pressure (BP) monitor, the developed prototype device reliably captured variations in carotid local ΔP induced by an external intervention.

**Conclusion:**

This technique could provide a direct measurement of local PWV, arterial dimensions, and a calibration-free estimate of beat-by-beat local ΔP. It can be potentially extended for calibration-free cuffless BP measurement and non-invasive characterization of central arteries with locally estimated biomechanical properties.

## Introduction

The prognostic importance of arterial blood pressure (BP) parameters as a risk factor for cardiovascular events is well established [1–3]. Among the parameters that characterize BP, the systolic BP (SBP) and diastolic BP (DBP) values are widely used as an independent predictor of future cardiovascular risks and outcomes. The numeric difference between SBP and DBP, referred to as pulse pressure (ΔP), is dependent on the cardiac output, elastic behavior of the aorta and large arteries, blood pulse wave reflection and pulse wave velocity (PWV) [4, 5]. Stiffer arteries are susceptible to rise in ΔP level due to the reduction in arterial compliance and associated increase wave reflection amplitude and PWV [6]. Physical stiffening of the large arteries is recognized as the consequence of chronic irreversible vascular aging and deposition of atherosclerotic plaques along the arterial walls. The prevalence of isolated systolic hypertension in middle-aged and elderly subjects is primarily due to the stiffening of the larger central arterial system [7]. Currently, there is increasing evidence that the risk factor of cardiovascular morbidity and mortality is higher when central aortic ΔP is larger for subjects with the same SBP level [8–12]. A precipitous increment in ΔP is widely observed after the age of 50–60 years [13], showing a direct association with end-organ damage, coronary heart disease, stroke, myocardial infarction and congestive cardiac failure, which are the leading causes of mortality. As alluded above, ΔP measurement from the central arterial system may be a recommendable physiological parameter to monitor as part of the routine clinical diagnostic practice and hypertension management.

Brachial artery SBP and DBP recorded using a pressure cuff by sphygmomanometry is routinely reported in standard clinical practice. The numeric difference between brachial SBP and DBP (brachial ΔP) is invariably higher than the ΔP measured from the central arteries, particularly in young subjects [9, 14]. The variation of pulsatile components of BP (ΔP and SBP) throughout the arterial tree is well explained using ‘pulse pressure amplification phenomenon’ [14]. Therefore, brachial ΔP measure is a poor surrogate for the corresponding central arterial ΔP level [15, 16], and these values respond differently to antihypertensive therapies and certain drugs [17, 18]. Direct use of conventional cuff-based devices for the measurement of BP parameters from the central arteries such as aorta and carotid is impractical. Tonometric devices based on the principle of applanation tonometry is the most commonly used non-invasive technique to estimate central BP (aortic or carotid) parameters. Calibration of carotid artery pressure pulse waveform with respect to separately measured brachial BP parameters is the basis of all tonometry-based central BP estimation methods [9, 19]. However, reliable acquisition of pressure pulse waveforms from the carotid artery using a tonometric sensor is particularly challenging, because it is difficult to effectively applanate the carotid artery individually, and erroneous signal recording may occur due to the movement of soft tissues at the carotid site owing to respiration [19]. Several clinical studies have reported the use of peripheral artery pressure pulse waveform (most devices recommend recording waveforms from the radial artery since it satisfies all conditions of a valid applanation tonometry) to estimate central BP parameters, using dedicated devices based on modified applanation tonometry and oscillometric principles. These devices rely on prediction models with best-case calibration strategies using manually or automatically measured brachial/radial BP to estimate the central BP parameters. Such scaling of peripheral pressure pulse waveform using mathematical transfer functions should be considered with caution [20, 21]. In addition to aforesaid methodological limitations, the current devices are operator-dependent and require skill to obtain quality recordings, which has hampered their widespread application. Therefore, a non-invasive technique for direct measurement of aortic/carotid BP parameters is essential, which could potentially enhance the interpretation of central arterial ΔP at the point of care for the prediction of future cardiovascular events.

In this work, we are demonstrating the design, verification, and validation of a novel method and system for cuffless, calibration-free evaluation of ΔP from the carotid artery (carotid ΔP). The developed system consists of a bi-modal arterial compliance probe which employs a pair of magnetic plethysmograph (MPG) sensors and a single-element ultrasound transducer for direct measurement of various physiological parameters from the carotid site. Design features of the bi-modal arterial compliance probe, associated electronics hardware, signal processing, and measurement algorithms are presented in the following sections. The principle of ΔP measurement using the proposed bi-modal sensor arrangement was initially demonstrated on an arterial flow phantom replicating blood flow through the human carotid artery. The measurement system could evaluate ΔP over continuous cardiac cycles from the recorded physiological parameters using established biomechanical equations, without any subject- or population-specific calibration. Feasibility of the proposed technique for real-time measurement of carotid ΔP in a continuous (beat-by-beat) manner was experimentally validated on 22 subjects under two different physical conditions: (1) Physically relaxed condition, (2) Post-exercise condition. The study results and observations are discussed in detail, which illustrate the potential of the proposed cuffless approach for carotid ΔP measurement without any calibration procedure.

## Materials and methods

### Principle of calibration-free cuffless ΔP measurement

**Fig 1:**
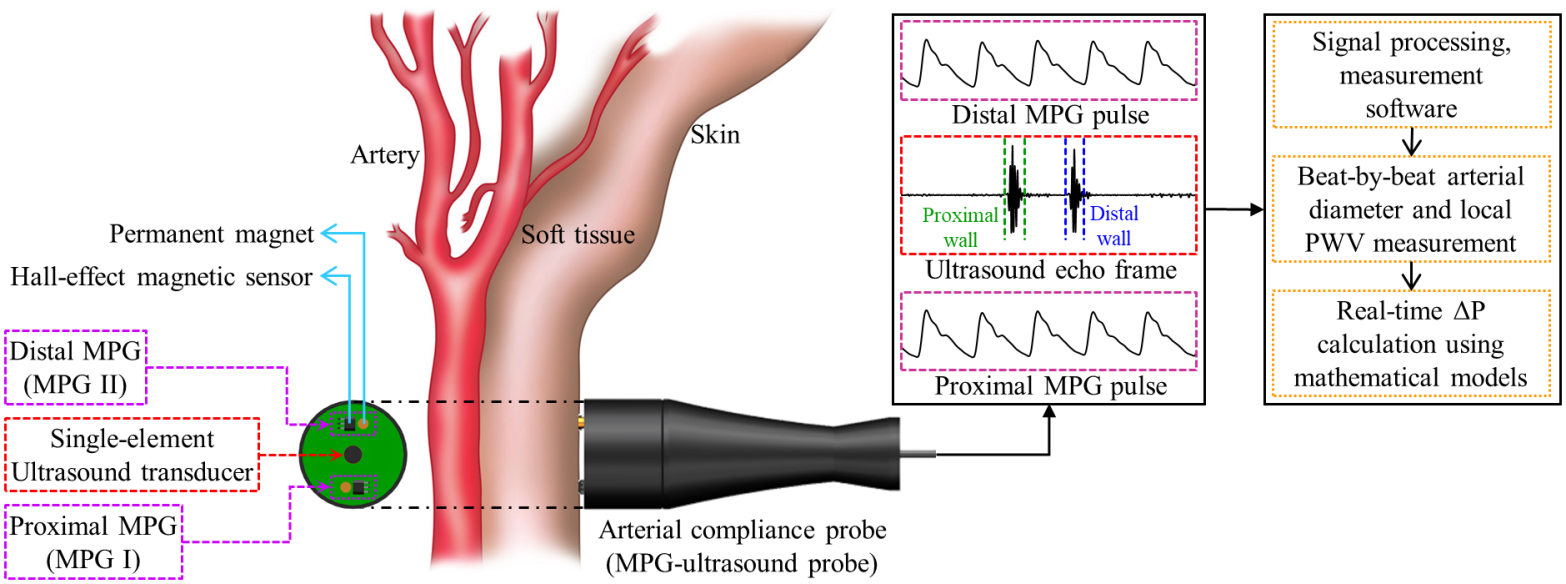
The principle of calibration-free cuffless local ΔP measurement from superficial arteries using arterial compliance probe.

Arterial wall properties and blood pulse transit time estimates within a small section of an artery (defined as ‘local indices’) change acutely as a function of BP at the measurement site. It is feasible to quantify the stress within a unit length of an artery in response to an applied ΔP using the variations in geometrical characteristics of the vessel and characteristic PWV [4]. Accordingly, a cuffless, calibration-free approach for ΔP measurement was introduced by identifying the relationship between locally measured ΔP, arterial dimensions, and PWV. As illustrated in Fig 1, a method and system with application-specific sensing modality and controller unit were developed to perform measurements from superficial arteries. The system included a hand-held probe configured to be held on the skin surface near an arterial site of interest. The probe comprised of dedicated sensing elements to simultaneously acquire local PWV and arterial dimensions such as arterial distension (ΔD) and end-diastolic lumen diameter (D_D_) over continuous cardiac cycles. These physiological parameters were directly used in a mathematical model for beat-by-beat cuffless ΔP evaluation.

The arterial wall dynamics associated with the pulsatile flow of blood (an incompressible viscous fluid) can be modeled using the Moens-Korteweg equation [22]. According this, Young’s elastic modulus (E) of an arterial section (vessel diameter = D, wall thickness = h) encompassing the pulsatile flow of blood volume with a mass density ρ and transit velocity equal to PWV can be expressed as given in (1).

$$E=\frac{\rho \;D{\;PWV}^{2}}{h}$$ (1)

Eq (1) was further modified by Bramwell and Hill [5] in relevant to non-invasively measurable physiological parameters that model the relation between local PWV and distensibility (κ) of the arterial wall [23], given as follows:

$$PWV=\frac{1}{\sqrt{\rho \;\kappa }}$$ (2)

The distensibility can also be evaluated using end-diastolic luminal area of the vessel (A_D_) and relative change in luminal area from diastolic phase to systolic phase (ΔA) due to an applied local ΔP during a cardiac cycle [23], and can be expressed as:

$$\kappa =\frac{\Delta A}{A_{D}\;\Delta P}=\frac{2\;\Delta D\;D_{D}+{\Delta D}^{2}}{\Delta P\;D_{D}^{2}}.$$ (3)

Substitution of (3) into (2) with rearrangement results in the local ΔP as a function of arterial wall properties and local PWV as illustrated in (4).

$$\Delta P=\rho \;{PWV}^{2}\;(2\frac{\Delta D}{D_{D}}+{\left(\frac{\Delta D}{D_{D}}\right)}^{2})$$ (4)

From (4), local ΔP can be estimated without any calibration procedure whenever ΔD, D_D_, and local PWV are simultaneously measured from a small arterial segment. It may be remembered that the normal human blood density ρ is typically in the range of 1040 kg/m^3^ to 1070 kg/m^3^ [24, 25]. Consistent with previous studies [23, 26], a fixed value ρ = 1060 kg/m^3^ was used in the present system in (4) for ΔP calculation. Therefore, an error of less than 1.88% associated with the variability in ρ across individuals is expected in ΔP level.

### Measurement system overview

#### Arterial compliance probe

A bi-modal arterial compliance probe was developed to simultaneously measure the essential variables of (4) for local ΔP evaluation. The initial design of the probe was optimized to perform measurements from the carotid artery in the neck, since it closely represents the central aortic conditions. As illustrated in Fig 1, the probe consisted of two independent sensing modalities: (1) a custom single-element ultrasound transducer (center frequency = 5 MHz) operated in the pulse-echo modality, and (2) identical MPG sensor pair, one on either side of the ultrasound transducer. This integrated arrangement with circular sensing area (diameter = 30 mm) was ideal for simultaneous measurement of desired physiological signals from the carotid site. The diameter parameters (ΔD and D_D_) were evaluated in real-time by acquiring strong and sharp ultrasound echoes from the arterial walls, by orienting the probe along the arterial diameter chord [27–30]. As shown in Fig 1, each MPG sensor unit consisted of a permanent magnet (D032A –Amazing Magnets LLC) and a highly sensitive Hall-effect magnetic sensor IC (SS49E T3 –SEC Electronics Inc.), which produces an output voltage proportional to the propagating blood pulse volume [31, 32]. Blood pulse waveforms from the proximal and distal MPG sensors (center-to-center separation distance = 23 mm) were continuously captured for real-time local PWV measurement.

#### Hardware architecture of the prototype device

A portable device that operated in conjunction with a Microsoft Windows^®^-based tablet computer (Acer Aspire P3) was developed. The device hardware include all sections required to excite the transducer/sensing elements and continuously acquire the response and output physiological signals. An industrial-grade microcontroller (ARM cortex-M4 –NXP semiconductor) was used to control the transmission and receiving modes of the ultrasound sensor, generation of short duration high voltage (±40 V) excitation pulses using a high-speed pulser IC (STHV748 –ST Microelectronics), and electronics modules to capture the ultrasound echoes from the measurement site. The received electrical signals were initially passed through low noise amplifier and active bandpass filters with a cutoff frequency of 1 MHz to 8 MHz. Dedicated on-board analog-to-digital converter (sampling rate = 80 MS/s, resolution = 12-bit) was used to digitize and sent the pre-processed ultrasound echo signals to the tablet computer via HSUSB 2.0 communication channel.

Electronics circuit of the local PWV module comprised of a dual-channel analog front-end (AFE) with negligible inter-channel delay. Hall-effect ICs of the arterial compliance probe was connected to the AFE using shielded cables and operated over a single 5V supply voltage. Raw MPG signals from the Hall-effect ICs contain a pulsatile physiological signal (in units of mV) superimposed on the quiescent offset voltage of IC. Specially designed subtractor circuits were used to remove the instantaneous quiescent offset voltage to extract the pulsatile components from raw MPG signals [31]. The acquired MPG signals from both the proximal and distal sensors were amplified (gain ≈ 40 dB) using a dual channel instrumentation amplifier (INA2126P—Texas Instruments). Digitized signals (using NI USB-6002 OEM—National Instruments; sampling rate = 25 kS/s/ch) were sent to the tablet computer in real-time along with the ultrasound echoes.

#### Measurement software architecture

A virtual instrumentation program with a custom graphical user interface (GUI) developed in National Instruments’ LabVIEW platform (measurement software) offered full control over signal acquisition/processing, simultaneous measurement of local PWV, ΔD, D_D_, and real-time local ΔP evaluation over continuous cardiac cycles. The acquired ultrasound echo frames and dual channel MPG signals were processed simultaneously, but independently in the digital domain. Construction of the arterial lumen diameter waveform from the ultrasound echo frames was performed using the fully automated algorithm of our clinically validated ARTSENS^®^ technology, demonstrated in a series of publications [27–29, 33–35]. Briefly, in this technology locations of arterial near and far wall in the filtered ultrasound echoes were identified using wall detection algorithm [28]. Displacement of the identified wall locations was continuously tracked in successive frames using a correlation-based motion tracking technique [28], that enables capturing of the arterial diameter waveform, absolute ΔD and D_D_ (in units of mm) in a beat-by-beat manner. Since the proposed approach is an image-free ultrasound technique [27–29], the quality of acquired ultrasound echoes was inspected continuously and displayed on the GUI by an application-specific signal quality parameterization algorithm [35].

MPG pulse waves from the proximal and distal sensors were initially filtered using a 2^nd^ order zero phase-shift Butterworth low-pass filter (cutoff frequency = 10 Hz). A cycle cutting algorithm was then applied in both the channels with equal time-window to segregate proximal and distal cycle pairs with their absolute time stamp. Further, the first derivative maximum technique was applied to measure local pulse transit time [36], and hence to evaluate local PWV using the fundamental distance-time equation (in units of m/s) in a beat-by-beat manner. Simultaneously measured local PWV, ΔD, and D_D_ were directly substituted into (4) to calculate the absolute local ΔP level (in units of mmHg) in real-time.

Erroneous estimation of local ΔP may occur during the real-time assessment due to non-ideal values of local PWV, ΔD, or D_D_ caused by breathing artifacts, unstable probe to skin attachment and/or degraded signal quality. These non-ideal effects were not considered in the blood pulse wave analysis algorithm. Therefore, a real-time screening test was performed on measured beat-by-beat local PWV, ΔD and D_D_ values before the final calculation, in order to discard all non-ideal values and corresponding pulse cycles. The occurrence of non-ideal values was indicated in real-time—this assisted the operator to re-orient the arterial compliance probe to acquire waveforms with desired signal quality.

A detailed discussion on the design features of arterial compliance probe, device hardware, measurement software architecture and signal processing algorithms can be found in S1 Appendix.

### Principle verification: Phantom study

#### Experimental setup

**Fig 2:**
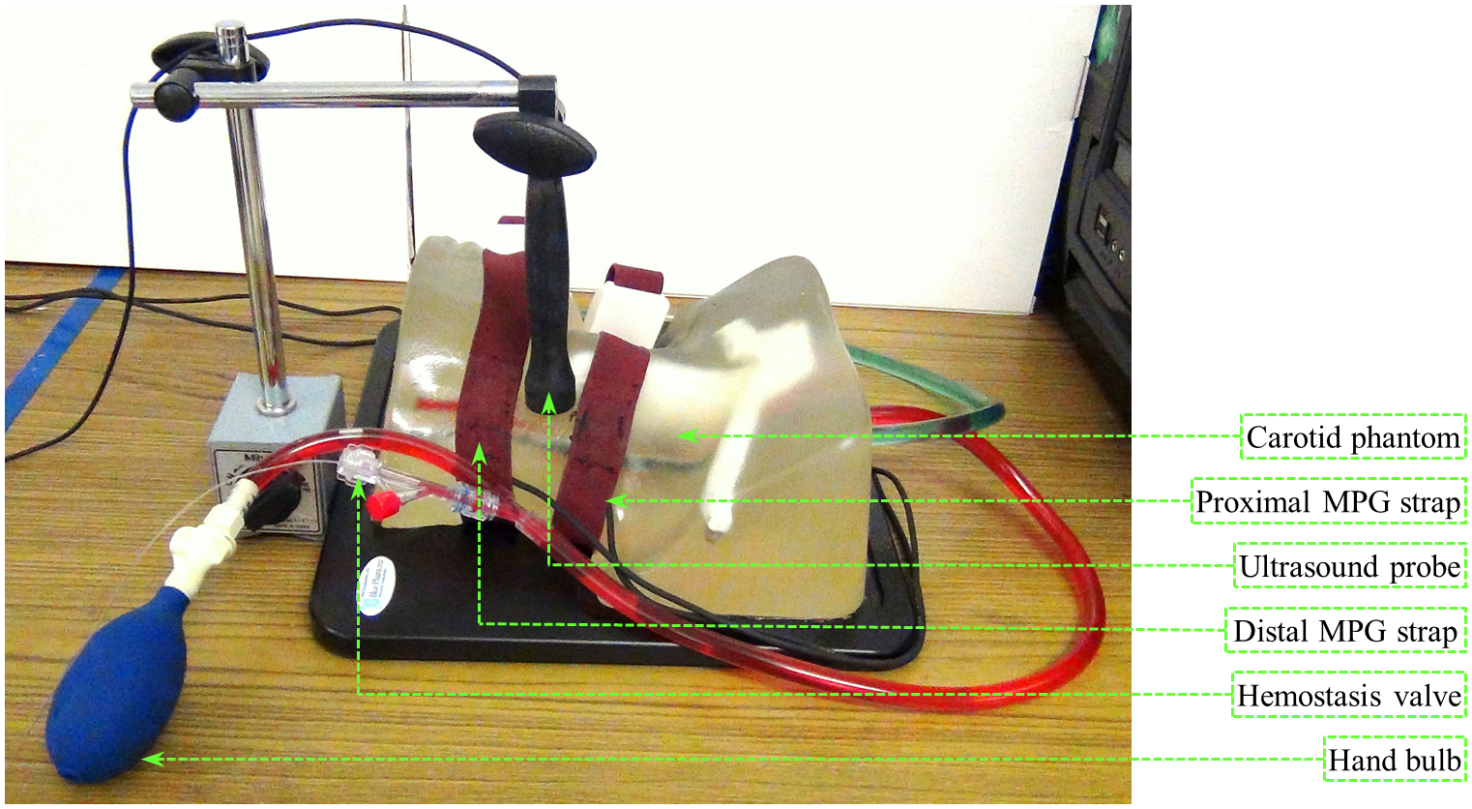
Experimental setup used for the phantom study to verify the proposed technique.

A commercially available arterial flow phantom (BPIJ500-C—Blue Phantom-CAE Healthcare) with an anatomical imitation of the human neck was preferred for verification of the proposed ΔP measurement principle. Experimental setup and placement of various sensing elements on the phantom have been illustrated in Fig 2. In this study, the integrated dual MPG-ultrasound arrangement of the arterial compliance probe was replaced with two identical MPG straps [37] and a single-element ultrasound probe for reliable and continuous measurement from the carotid phantom. The straps were wrapped around the phantom neck (center-to-center distance = 50 mm) by keeping both the sensing elements above the internal elastic which tube emulates human carotid artery. Further, the ultrasound probe was placed between the proximal and distal MPG straps, resembling the bi-modal arterial compliance probe. A calibrated high-fidelity invasive pressure catheter (SPR-882 –Millar Instruments Inc.) was inserted into the elastic tube through a hemostasis valve to record the true internal ΔP as the reference measurement.

#### Data collection

Pulsatile flow of the blood mimicking fluid through the elastic tube was manually simulated at various pressure levels and pulse rates by controlled, repetitive pumping of the provided hand bulb. Corresponding variations in diameter parameters (ΔD and D_D_) and pulse propagation velocity (equivalent to local PWV) were continuously captured using the developed system. Real-time pulse-by-pulse ΔP was calculated with the measured local PWV and diameter parameters—henceforth referred to as estimated ΔP. The reference arterial pressure waveform from the pressure catheter was acquired via ADInstruments’ Bridge Amp and PowerLab interfaced to the LabChart software. A custom program was executed in the LabChart software to evaluate pulse-by-pulse reference ΔP by taking the difference between the maxima and minima of continuous arterial pressure cycles. Presence of an outlier/non-ideal value in the measured variables or reference value led to the real-time exclusion of the corresponding pulse cycle from further computation and statistical analyses. Data recorded using the prototype device for the present study is available in S1 Dataset.

### Prototype validation: In-vivo study

#### Participants

Experimental validation of the proposed cuffless, calibration-free local ΔP measurement method was performed through an in-vivo study in twenty-two normotensive subjects (N = 22; 15 males and 7 females, mean age = 24.5 ± 4 years, mean body mass index (BMI) = 22.7 ± 3.2 kg/m^2^). The group selected for this study comprised of employees from our research laboratory (Healthcare Technology Innovation Centre (HTIC), IIT Madras Research Park, Chennai, India)–they were recruited in an opportunistic manner. All the recruited subjects volunteered to participate in this study. No benefits were offered to the study participants. They were instructed to abstain from caffeinated beverages and strenuous exercise for 24 hours. Participant exclusion criteria were suspected cardiac conditions, intake of any long-term medications, regular exercise habit, and athletic involvement.

#### Study protocol and data collection

The study protocol was reviewed and approved by the review committee of HTIC, IIT Madras Research Park, Chennai, India, and the procedures were performed in accordance with the guidelines of the review committee. This study was carried out in compliance with the Helsinki Declaration of 1975, as revised in 2000. The total duration of the study was seven working days (October 7–15, 2015), performed in morning hours (9:00 a.m.–11.30 a.m.) with an average of three subjects per day. All the procedures were explained and written informed consent was obtained from each participant. The individual in this manuscript has given written informed consent (as outlined in PLOS consent form) to publish the photographs and case details.

**Fig 3:**
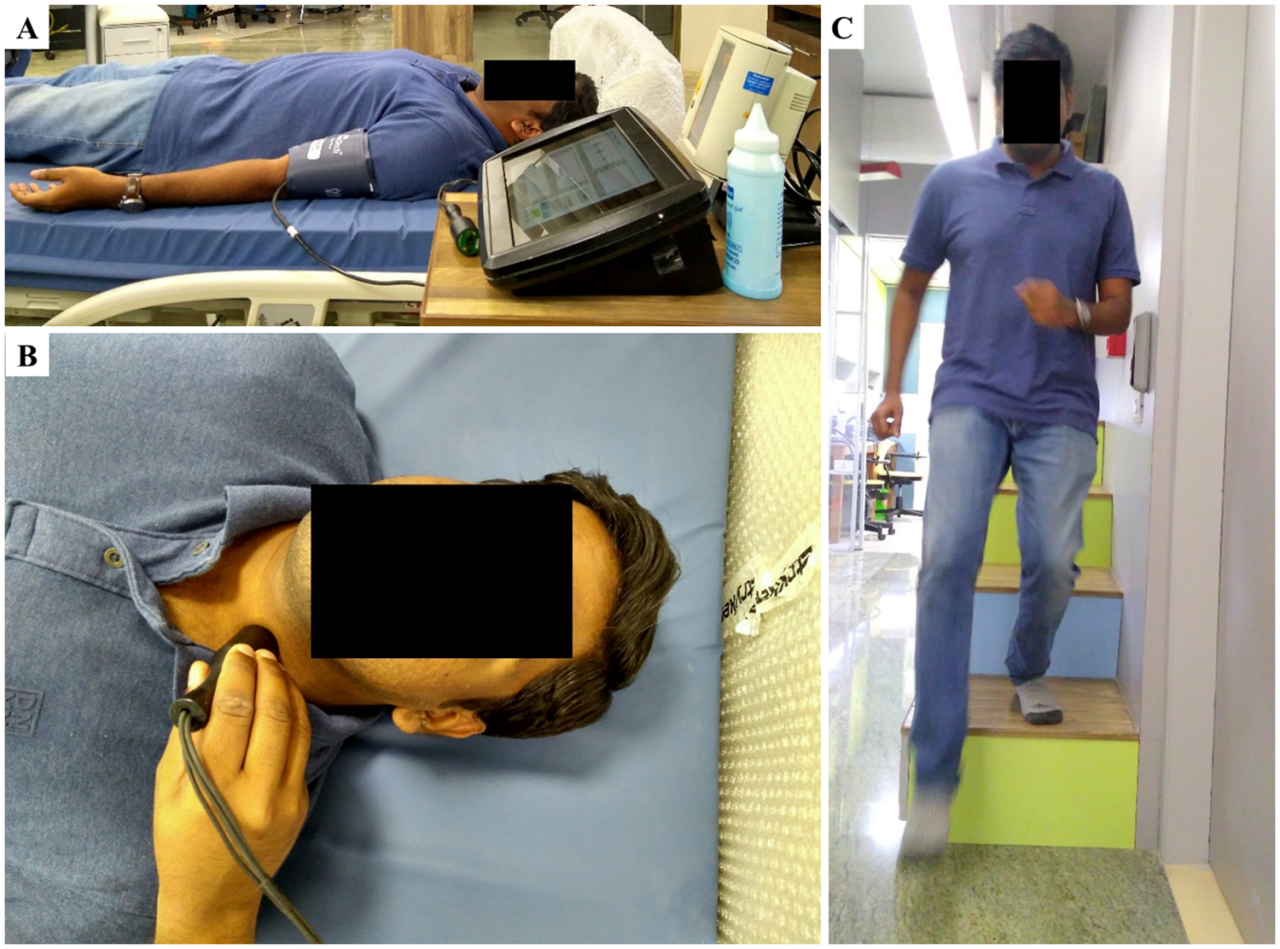
In-vivo validation study protocol. (A) In-vivo study setup; showing brachial BP measurement from the left hand. (B) The operator performing local ΔP measurement from the left carotid artery. (C) Subject performing stair-running exercise.

The in-vivo validation study comprised of real-time measurement of beat-by-beat local PWV, arterial dimensions, local ΔP (from the carotid artery), and reference BP parameters (from the brachial artery) under two different physical conditions; (1) physically relaxed condition, and (2) post-exercise condition. Measurements under different physical conditions were performed to confirm that the proposed device could capture variations in local ΔP due to modulations in the hemodynamic mechanism. All the measurements were recorded by a single operator with subjects positioned supine on a hospital bed. Photographs shown in Fig 3 illustrate the study protocol. Initially, all the participants rested for 5–10 minutes to stabilize their heartrate and BP level—this allowed to achieve measurements under physically relaxed condition. Brachial BP parameters were obtained using an automated clinical grade BP monitor (SunTech^®^ 247™ –SunTech Medical) with a pressure cuff on the left upper arm (Fig 3A). As shown in Fig 3B, arterial compliance probe was then placed on the neck (left-side) after locating the carotid pulse by palpation. The operator adjusted the probe placement and orientation on the neck over the carotid artery such that strong ultrasound echoes from arterial walls and dual MPG signals were simultaneously received. Local PWV, arterial dimensions, and carotid local ΔP were continuously measured for 30–60 seconds.

After resting for one more minute, subjects performed a stair-running exercise (through a four-stepped staircase with 1.0 m total rise, shown in Fig 3C) for approximately 2 minutes. The brachial pressure cuff was detached from the BP monitor and kept on the arm at the same position while performing the exercise to avoid cuff bladder positioning error in post-exercise BP measurement. Immediately after completing the exercise (continuous run for 2 minutes or until reaching an individual’s exercise tolerance level), post-exercise data (brachial BP parameters, carotid local PWV, arterial dimensions, and local ΔP) was measured in supine posture by following the same procedures.

### Statistical analysis

Continuous physiological parameters are expressed as mean ± standard deviation (SD). An indicator ‘beat-to-beat variation’ was introduced to assess their variability over continuous beats under physically relaxed condition; which is defined as the ratio of SD to mean of finite consecutive measurements (exclude non-ideal values if any) expressed in percentage. Beat-to-beat variation during the post-exercise recovery period was not considered in the present study since all the measured parameters were steadily varying to recover the stable baseline values. Box-and-whisker diagrams are used to demonstrate the grouping and variability of the measured parameters. The Bland-Altman analysis was performed to study inherent trends and degree of variation between the measurements from two independent systems. The agreement between two absolute measurements was investigated via linear regression analysis and expressed using Pearson’s correlation coefficient (r), a value of p < 0.05 was considered statistically significant.

## Results and discussion

### Verification using arterial flow phantom

**Fig 4:**
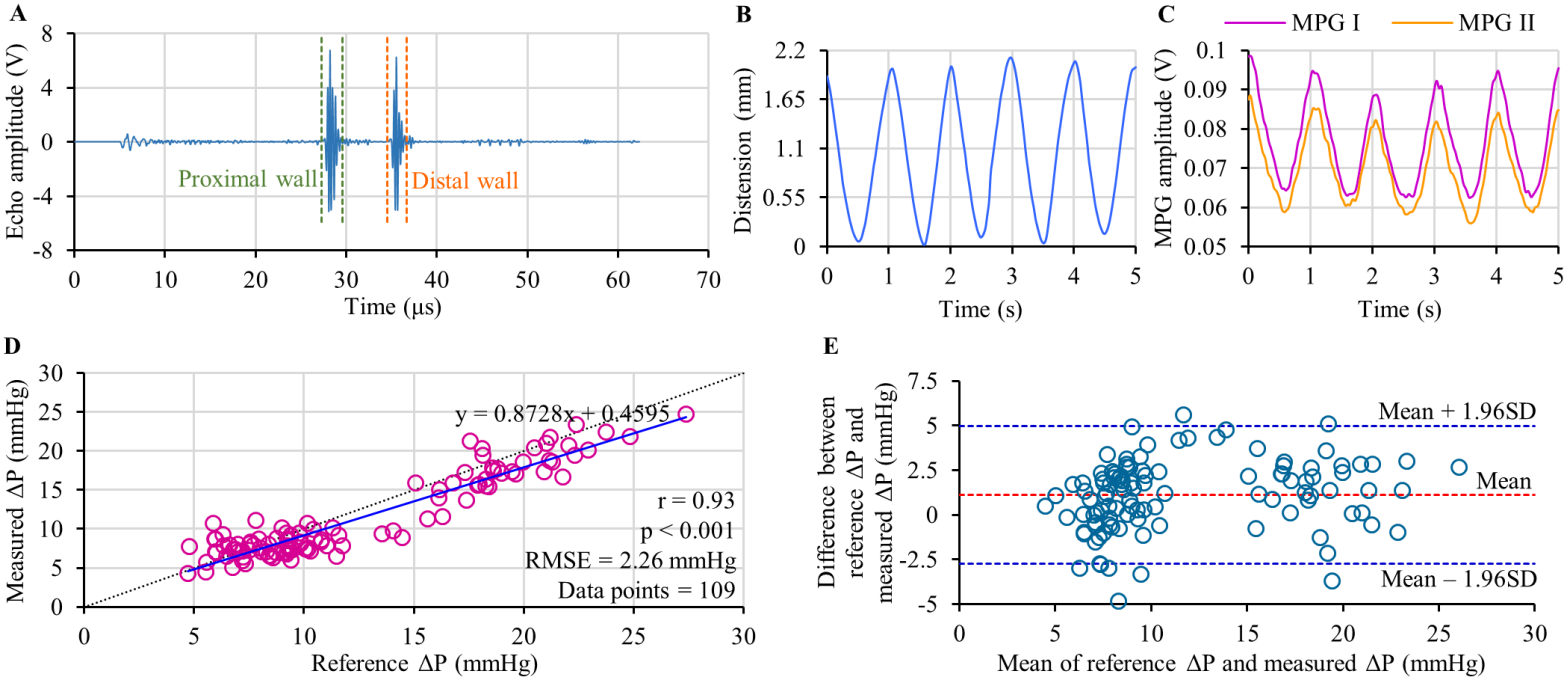
Summary of phantom-based verification study results. (A) Ultrasound echo frame from the carotid phantom. (B) A sample of continuous distension wave. (C) A sample of dual MPG waveform acquired along with the distension wave. (D) Correlation analysis between reference ΔP from catheter and ΔP estimated using the MPG-ultrasound arrangement. (E) Bland-Altman analysis showing the degree agreement between the reference and estimated ΔP.

The results and observations from the phantom-based experimental verification study have been summarized in Fig 4A–4E. The developed system continuously captured high-fidelity ultrasound echoes and dual MPG waveforms from the arterial flow phantom. Typical ultrasound echo signal obtained from the measurement site of the phantom is depicted in Fig 4A. Both proximal and distal walls of the elastic tube are clearly defined in the echo signal frame. The instantaneous out-of-phase motion of these wall echoes was efficiently tracked by the measurement system over consecutive pulse cycles to obtain the arterial lumen diameter waveforms. A sample of the flow phantom distension waveform (arterial diameter changes ΔD plotted as a function of time) is shown in Fig 4B. Sample waveforms of dual MPG pulse cycles (MPG I—proximal MPG cycle and MPG II—distal MPG cycle) recorded from the adjacent measurement sites are shown in Fig 4C. The amplitude of raw MPG signals from the phantom was approximately 0.25 mV– 0.5 mV. Characteristics of MPG signals were identical to the distension waveform with equal pulse rate.

In the scatter plot in Fig 4D pulse-by-pulse reference ΔP values is compared against estimated ΔP values pooled over continuous phantom pulse cycles simulated at various pressure level. The regression analyses of reference ΔP versus estimated ΔP revealed a statistically significant r-value equal to 0.93, with p < 0.0001. Resultant regression line y = 0.8728x + 0.4595 has been shown in the same figure (solid line), which is close to the ideal regression line y = x (dotted line). Root-mean-square-error (RMSE) in ΔP measurement from the phantom using the developed system was 2.26 mmHg. In confirmation of the accuracy of the proposed technique, it may be observed from the Bland-Altman analysis (Fig 4E) that the estimated values agreed with the reference measurements. A non-significant mean bias of 1.11 mmHg, limits of agreement of –2.75 to 4.98 mmHg and SD of error (reference ΔP—estimated ΔP) equal to ±1.97 mmHg were reported. Results obtained from this phantom-based study verified the principle of cuffless, calibration-free ΔP estimation using the ultrasound-MPG arterial compliance probe. This bi-modal sensor arrangement and measurement system were further validated through a formal non-invasive human study using arterial compliance probe and the developed prototype device.

### In-vivo experimental validation

#### Reliability of signal acquisition from human subjects

**Fig 5:**
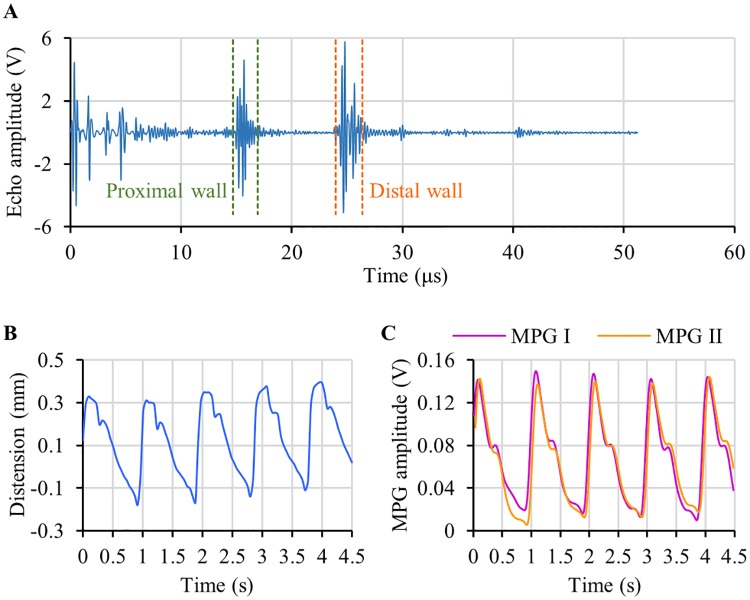
Sample waveforms recorded from human carotid artery using the arterial compliance probe. (A) An ultrasound echo frame with proximal and distal arterial walls (B) A sample of arterial distention waveform, and (C) simultaneously acquired dual MPG waveforms.

The in-vivo experimental study protocol was completed in all participants, and the desired physiologic signals/measurements were recorded under two different physical conditions. Real-time signal quality indicator and visual feedback allowed the operator to acquire high-fidelity ultrasound echo signals (signal-to-noise ratio ≈ 28 dB) from the carotid artery, in which both the proximal and distal arterial walls were clearly defined. Typical echo frame obtained from the left common carotid artery of a subject (age = 27 years, BMI = 26.15 kg/m^2^, baseline SBP/DBP = 123/79 mmHg) after initial signal processing (refer S1 Appendix) has been shown in Fig 5A. Developed measurement algorithm was efficient in automatically distinguishing the arterial proximal and distal wall echo from other static echoes. The current ultrasound frame rate of 82 Hz was sufficient for reliable tracking of instantaneous displacement in arterial wall echoes and construction of lumen diameter waveforms. A sample of the carotid artery distension waveform obtained from the same subject is shown in Fig 5B. Echo tracking and diameter measurements were feasible even when the heartrate and BP level increased after performing the physical exercise.

A sample of carotid proximal and distal site MPG waveforms (MPG I and MPG II respectively) acquired along with the distension waveform of the aforesaid subject has been presented in Fig 5C. The MPG waveform characteristics were subjective and attributed to an individual’s physical conditions. In the current probe design, the peak-to-peak amplitude of raw carotid MPG signals varied approximately between 1.15 mV_PP_ to 3.5 mV_PP_. The carotid MPG signals acquired under post-exercise condition possess approximately 9%– 16% higher peak-to-peak amplitude compared to the signals acquired under physically relaxed condition. Relatively low peak-to-peak amplitude was observed from the subjects with obesity and/or mild carotid surface pulsations. However, local PWV evaluation is independent of the MPG signal amplitude because only the timing information from the systolic region is used in the measurement technique (refer S1 Appendix). Carotid artery MPG signal quality achieved with the present prototype system (signal-to-noise ratio ≈ 30 dB) was sufficient for resolving the time delay between proximal and distal waves (acquired 23 mm apart) to calculate local PWV. Despite the need of moderate level operating skill to orient the arterial compliance probe at the measurement site (positioning of all three sensors above the arterial vessel), continuous acquisition of ultrasound echoes and dual MPG signals using the prototype device was near operator-independent and largely guided by the fully automated measurement software.

#### Real-time measurements from the carotid artery

**Fig 6:**
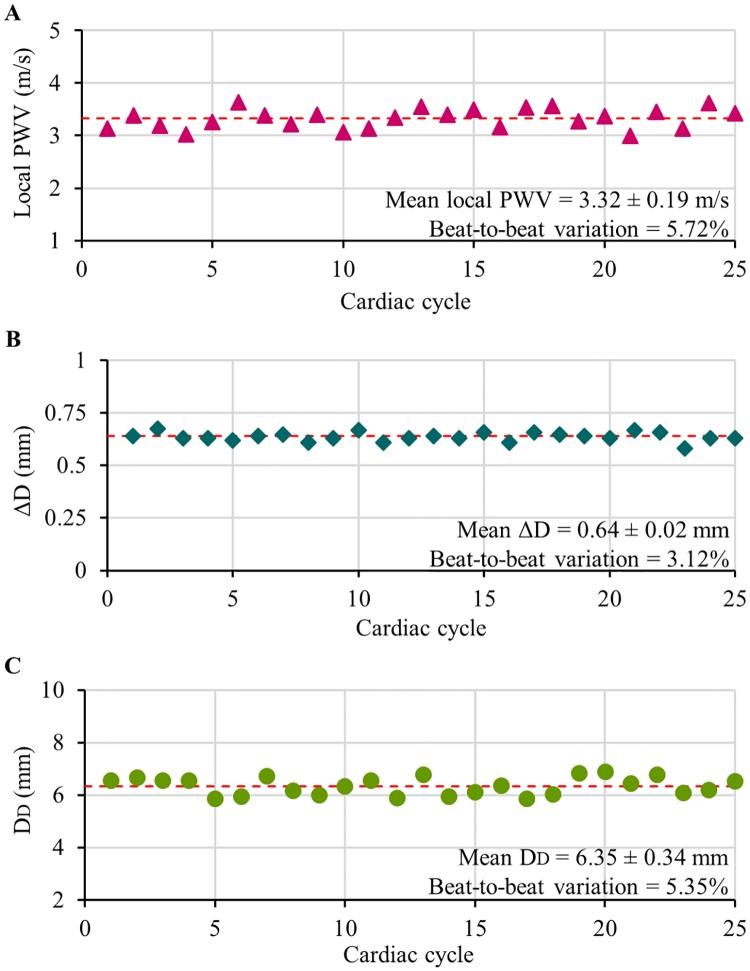
Simultaneously recorded beat-by-beat physiological parameters from the carotid artery. (A) Local PWV, (B) arterial distension ΔD, and (C) end-diastolic diameter D_D_.

**Fig 7:**
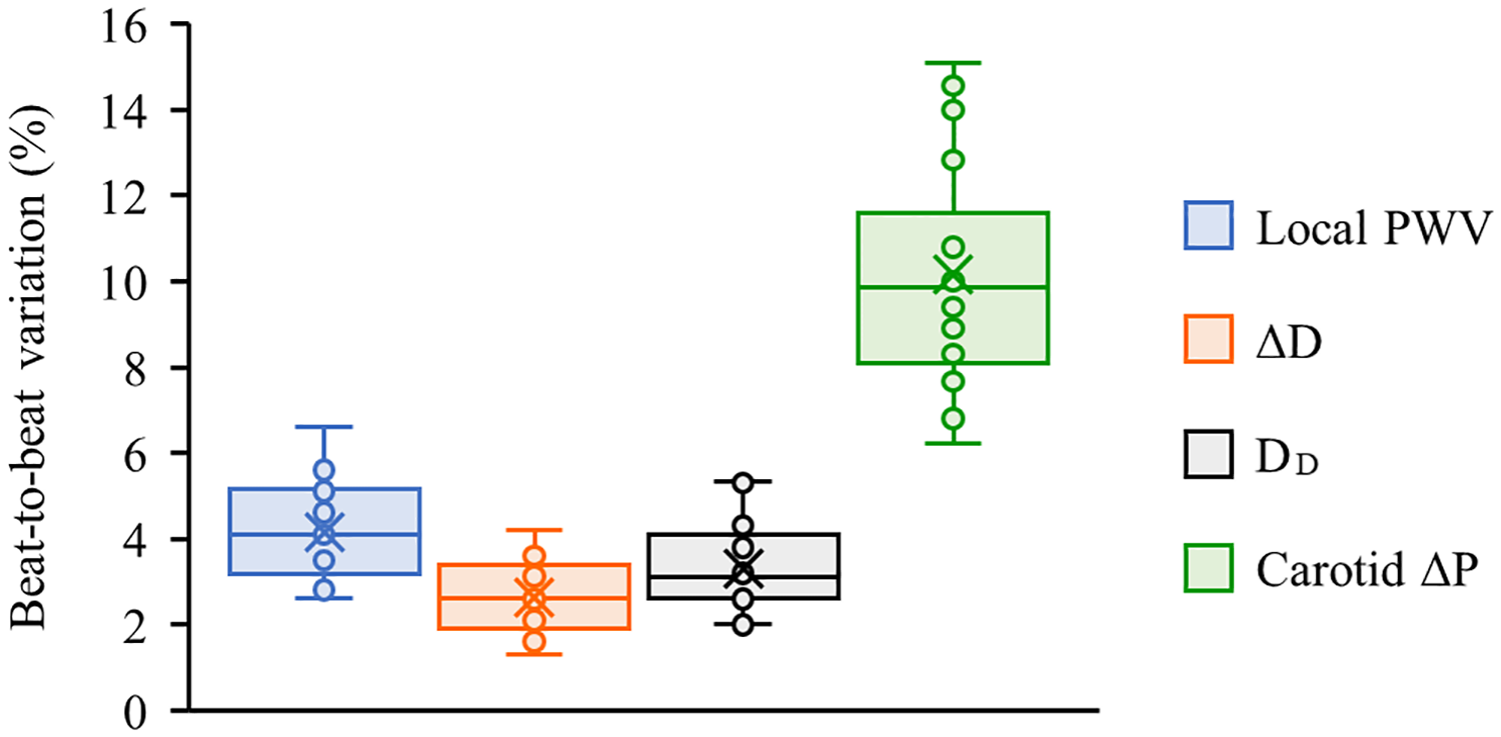
Beat-to-beat variations in continuous physiological parameters. Box-and-whisker diagrams showing beat-to-beat variation in the measured local PWV, ΔD, D_D_, and estimated carotid ΔP under physically relaxed condition (data pooled over all the subjects).

Fig 6A–6C shows the plots of simultaneously measured beat-by-beat local PWV, and ΔD and D_D_ from the carotid artery of a subject (age = 25 years, BMI = 25.11 kg/m^2^, baseline SBP/DBP = 110/68 mmHg) under physically relaxed condition. The measurements were found to be repeatable (beat-to-beat variation < 6%) over continuous cardiac cycles. Similar performance was reported from all the recruited subjects. Box-and-whisker diagrams shown in Fig 7 exhibit the beat-to-beat variation in carotid local PWV, ΔD, and D_D_ under physically relaxed condition pooled over all the subjects. The observed beat-to-beat variation in local PWV, ΔD and D_D_ measurement was less than 6.6%, 4.2%, and 5.3% respectively. It may be noted that the ultrasound-based measurements (direct estimation from the artery) were more repeatable than the local PWV estimated using MPG sensors (measured from the skin surface). Higher repeatability and reproducibility of local PWV measurements attributed to the practical limitations in getting good quality blood pulse signals while positioning the arterial compliance probe over the carotid artery.

The group average ΔD and D_D_ were 0.57 ± 0.09 mm and 5.54 ± 0.76 mm respectively, the normal range for the recruited age category. The carotid local PWV values (group average = 3.31 ± 0.49 m/s) were less than the typical range of regional PWV assessed from larger arterial sections and muscular arteries [38]. Reduced values of local PWV was consistently observed in our previous studies [31, 39, 40]. This is also reported by independent researchers working on PWV measurement from elastic arteries [38, 41]. It may be remembered that the physical and physiological characteristics of blood pulse propagation through an arterial segment is determined by its functional and structural factors such as BP level, vascular tone, and vascular hypertrophy. Thus, regional PWV assessed from larger segments such as the carotid-to-femoral arterial segment or carotid-to-radial arterial segment (heterogeneous structure with different mechanical characteristics) are typically higher in magnitude than the local PWV obtained from a small section carotid artery (a cushion artery).

**Fig 8:**
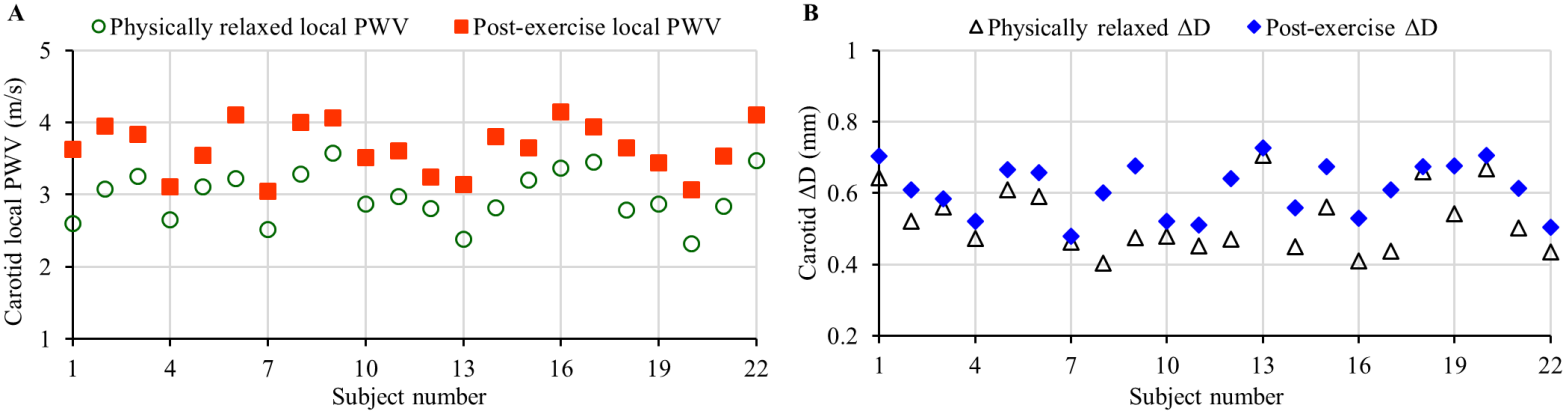
Mean carotid local PWV and ΔD of each individual measured from both the physically relaxed and post-exercise conditions.

As expected, the stair-running exercise induced a transient change in the hemodynamic parameter for all the recruited subjects. The degree of increment in SBP and ΔP was higher than the DBP level due to the aerobic nature of the exercise [42], the corresponding changes in local PWV and arterial dimensions were reliably captured using the developed arterial compliance probe. Fig 8A and 8B respectively exhibit the mean carotid local PWV and ΔD (mean of 5–10 consecutive cardiac cycles after removing outliers, if any) of each subject under physically relaxed and post-exercise conditions. A significant increment in local PWV from the baseline value was observed in all subjects (Fig 8A) after performing the exercise (group average post-exercise increment in local PWV = 0.67 ± 0.18 m/s; mean percentage increment = 22.9%). The group average increment in ΔD was 0.08 ± 0.06 mm with a mean percentage increment of 18.2% (Fig 8B). However, no significant change in the D_D_ values was observed after the exercise (group average variation in D_D_ < 2.8%). The post-exercise recovery time of local PWV, ΔD and other hemodynamic parameters such as BP and heartrate to regain their respective baseline value was subjective and depended on exercise intensity. These results readily demonstrated the efficiency of the proposed system in capturing BP induced variations of local PWV and arterial dimensions under different physical conditions.

#### Calibration-free cuffless evaluation of carotid local ΔP

For each beat, the developed measurement system calculated carotid ΔP in real-time using simultaneously measured local PWV and arterial dimensions without any subject- and/or population-specific calibration. Non-invasive local ΔP measurements were repeatable with a group average beat-to-beat variation of 10.2%–this was satisfactory for the current proof-of-concept prototype version. The observed beat-to-beat variation in carotid ΔP of individual subjects (under physically relaxed condition) has been presented in Fig 7 using the box-and-whisker diagram. Due to the squared term of local PWV in (4), its measurement accuracy significantly influences the beat-to-beat variation of estimated local ΔP than that of the arterial dimensions. Real-time carotid ΔP measurement quality was further quantified by introducing an error rate: the ratio of the total number of non-ideal cycles (acquired cardiac cycles with non-ideal values of local PWV or ΔD or D_D_) to the total number of acquired cycles. To examine the degree of error rate in the developed prototype device, carotid ΔP was recorded over continuous cardiac cycles (for a duration of 2.5–3 minutes) in supine posture from selected subjects (6 out of 22 subjects). The observed error rate in this study was less than 3.5%, illustrating the reliability of the proposed MPG-ultrasound arterial compliance probe in performing continuous measurement from the carotid site.

**Fig 9:**
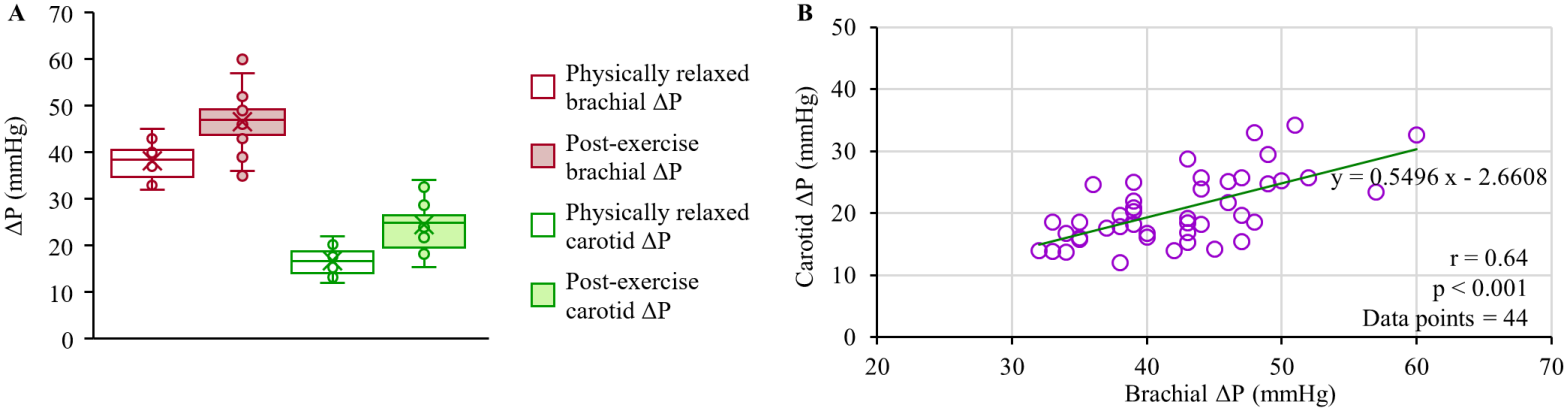
Reference brachial ΔP versus estimated carotid ΔP. (A) Box-and-whisker diagrams illustrating the brachial ΔP and carotid ΔP under physically relaxed and post-exercise conditions. (B) Correlation plot of brachial ΔP versus carotid ΔP.

It may be noted that the pulsatile components of the arterial pressure (SBP and ΔP) varies continuously throughout the arterial tree due to the phenomenon of pulse pressure amplification [14]. DBP level and mean arterial pressure (MAP) are relatively constant in the arterial system in the absence of hydrostatic effect. Therefore, brachial ΔP may not represent the absolute carotid ΔP level (carotid ΔP < brachial ΔP in the young and healthy population [9]), and hence their absolute values did not compare via Bland-Altman analysis in the present study. Fig 9A presents mean carotid ΔP and brachial ΔP recorded from the recruited subjects under physically relaxed and post-exercise conditions. Like the reference brachial BP monitor, the developed prototype device readily shows its ability to capture variation in baseline carotid ΔP level caused by an external physical intervention. Consistent with the physiological theory [8, 9, 14], the carotid ΔP for young subjects were on an average 22 mmHg lower than that of the reference brachial ΔP measurements. Furthermore, the linear regression analysis of carotid ΔP versus brachial ΔP performed using data consolidated from all the recruited subjects under both the physical conditions (Fig 9B; total data points = 44) revealed a statistically significant correlation with r = 0.64, p < 0.001, and a regression line y = 0.5496x−2.6608.

**Fig 10:**
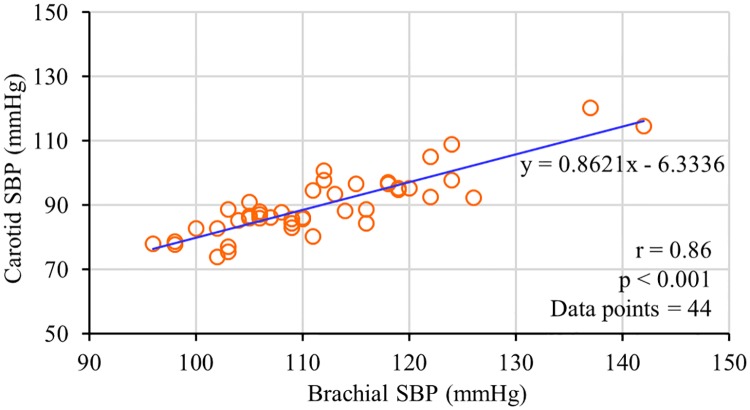
Correlation between measured brachial SBP and estimated carotid SBP (data pooled over all the subjects).

Considering the relatively constant nature of DBP level at brachial and carotid arteries while adopting the supine posture [9], SBP at the carotid artery was evaluated by numerically adding brachial DBP and carotid ΔP (carotid SBP ≈ brachial DBP + carotid ΔP; in supine posture). In Fig 10, estimated carotid SBP is plotted as a function of brachial SBP (data from both the physically relaxed and post-exercise conditions are pooled over all the subjects; total data points = 44). Both the measurements were correlated well with r = 0.86 and p < 0.001. The linear regression line y = 0.8621x−6.3336 (shown in Fig 10) illustrates that the carotid SBP level for the recruited normotensive population is 14% lower than the brachial SBP level; these results are consistent with the previous studies [43]. Although DBP level at both the carotid and brachial artery sites are not the same, their absolute difference is insignificant in supine posture due to a negligible hydrostatic difference. Therefore, carotid SBP obtained via this approach could potentially be a reliable estimate of pressure level at the central arteries. Since all the measurements were performed in a calibration-free manner, the obtained carotid BP parameters may thus be more accurate compared to conventional subject-specific calibration and/or pre-generated population-specific mathematical model-based central aortic BP evaluation techniques.

### Future research directions

The present study results and observations support the potential application of MPG-ultrasound arterial compliance probe to capture carotid local ΔP in a cuffless, calibration-free manner. However, the current prototype design still has limitations and future efforts are needed to bring this novel approach to practice. The major limitation concerns its usability. A moderate operating skill is required to orient all three sensors of the arterial compliance probe along a small arterial section, especially while performing continuous ΔP measurement from superficial arterial sites other than the carotid artery.

Current ongoing work includes the development of an optimized and advanced arterial compliance probe design with a provision for individual orientation adjustment for ultrasound and MPG sensors. An optimization of hardware parameters and acquisition module are also necessary to record ultrasound echoes at a higher frame rate to construct arterial diameters waveforms with high resolution. In-vivo validation on a large population by including different clinical BP categories from various age group is essential to further validate the proposed non-invasive, calibration-free, and cuffless ΔP monitor, and hence to demonstrate its potential clinical applicability. Future in-vivo studies also include extensive validation of the developed method against a real gold-standard reference device (invasive method using a pressure catheter) over patients undergoing catheterization.

Finally, the research and development work to realize a complete calibration-free technique for cuffless evaluation of BP parameters (SBP, DBP, ΔP, and MAP) is also in progress. A set of mathematical models were derived from the established relationships between arterial pressure and instantaneous variation in local PWV, as well as the non-linear relations between pressure and, the dimensional and material property variations of the arteries [44]. These calibration-free models can be directly used for cuffless BP measurement by simultaneously capturing the variation in local PWV, arterial dimensions, and material properties within a cardiac cycle. As discussed herein, the developed prototype device could reliably measure arterial dimensions and variation in lumen diameter (arterial distension). Recently, Nabeel et al. demonstrated the ability of dual-MPG sensors to capture carotid local PWV from distinct fiducial points within a cardiac cycle with high repeatability and reproducibility [45]. Necessary enhancements would be incorporated to the proposed MPG-ultrasound arterial compliance probe and measurement algorithms to simultaneously acquire all the desired physiological parameters (and their instantaneous variations) and thereby develop a calibration-free system for cuffless BP measurement and non-invasive characterization of central arteries.

## Conclusions

We have developed a bi-modal arterial compliance probe and built a physical device to perform non-invasive assessment of local ΔP from superficial arteries in a cuffless, calibration-free and beat-by-beat manner. The principle of the proposed technique was verified by in-vitro experiments using an arterial flow phantom with an anatomical imitation of the human neck, and the feasibility was demonstrated. A statistically significant strong correlation (r = 0.93, p < 0.001) was found for ΔP estimated from the phantom using the proposed technique when compared against the reference ΔP obtained from an invasive pressure catheter. Bland-Altman analysis of the estimated and reference ΔP from the phantom (simulated at various pressure level) yielded a non-significant bias of 1.11 mmHg and SD of error equal to ±1.97 mmHg. An in-vivo study was conducted on 22 young, healthy subjects to investigate the reliability and real-time cuffless, calibration-free ΔP evaluation capability of the proposed method. Local ΔP was measured from the left common carotid artery under physically relaxed and post-exercise conditions. The prototype device demonstrated the expected functionality and reliably captured beat-by-beat local PWV, ΔD, D_D_, and local ΔP from the human subjects under two different physical conditions. Measured carotid ΔP (without any calibration procedure) and carotid SBP level (estimated using brachial DBP and carotid ΔP) were found to correlate well with their corresponding brachial reference measurements with r = 0.64, p < 0.001 and r = 0.86, p < 0.001 respectively. The present study results support reliability and potential application of the dual MPG-ultrasound arterial compliance probe and associated measurement system for carotid ΔP (an indicator of central aortic ΔP) evaluation. Our initial prototype system was applied for carotid artery; however, this could be easily extended for real-time calibration-free evaluation of local ΔP, BP parameters, and various arterial stiffness indices from other superficial arteries.

## Supporting information

S1 Appendix Design of calibration-free cuffless pulse pressure monitor. Details of arterial compliance probe design, device hardware and measurements software design features.
(PDF)

S1 Dataset Data from prototype device. Dataset for the reported phantom-based verification and in-vivo experimental validation study.
(RAR)
