# Supplementary material for: Arterial compliance probe for cuffless evaluation of carotid pulse pressure
Source: PLoS One. 2018 Aug 16;13(8):e0202480. doi: 10.1371/journal.pone.0202480 (PMC6095577; doi:10.1371/journal.pone.0202480)
Supplement: S1 Appendix — Details of arterial compliance probe design, device hardware and measurements software design features. (PDF) [file pone.0202480.s001.pdf]

Arterial compliance probe for cuffless evaluation of carotid pulse pressure

Corresponding author:

Nabeel P M

Department of Electrical Engineering, Indian Institute of Technology Madras, Chennai, Tamil Nadu, India

E-mail: nabeelnpm@gmail.com, nabeelpm@ieee.org

Design of calibration-free cuffless pulse pressure monitor

Bi-modal arterial compliance probe design

A bi-modal arterial compliance probe was designed and developed to simultaneously measure the essential variables of (4) to enable cuffless and calibration-free evaluation of ΔP. The initial design of the probe was optimized to perform measurements from the carotid artery in the neck (carotid site). The carotid artery was preferred for local ΔP assessment using the initial prototype device since it is superficial and easily palatable, it closely represents the central aortic conditions and offers a straight, branch-free pathway till the bifurcation. However, this device could be easily extended for local ΔP measurement from other superficial arteries. S1 Fig exhibits the proposed initial prototype of the bi-modal arterial compliance probe. The probe consisted of two independent sensing modalities: (1) a single-element ultrasound transducer, operated in the pulse-echo modality for ΔD and D_D measurement, and (2) identical MPG sensor

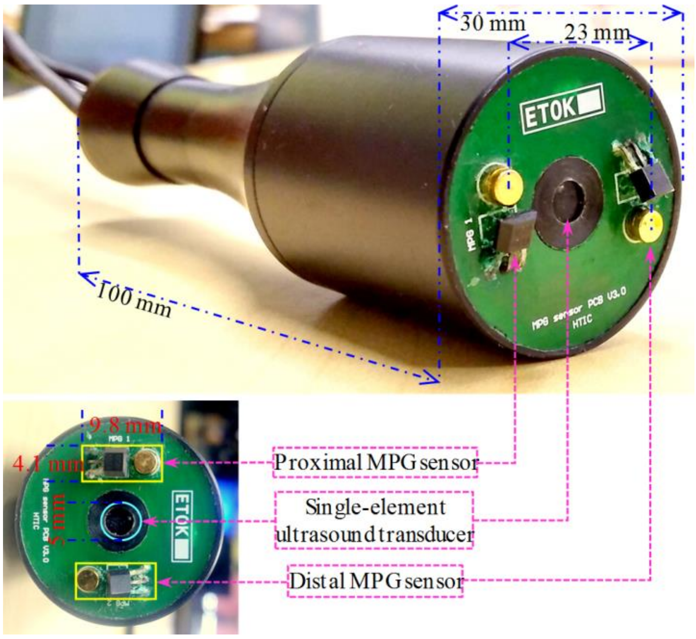

S1 Fig. The prototype design of the MPG–ultrasound arterial compliance probe.

pair, one on either side of the ultrasound transducer for local PWV measurement. A custom probe housing (total length = 100 mm) was machined using Delrin material with a circular transducer holder (diameter = 30 mm) for MPG sensor PCB, and a suitable mounting mechanism to fix the ultrasound transducer at the center. The proposed probe design and sensor arrangement allowed quick and reliable capture of physiological waveforms from multiple subjects with minimal operator dependence.

A custom-made piezoelectric ultrasound transducer (outer diameter = 5 mm) was fixed at the center of the probe (S1 Fig), for transmission of sharp ultrasound pulses at high frequency (center frequency = 5 MHz) into the carotid site. These sound pulses were reflected from various structures in their propagation path, including near (proximal) and far (distal) walls of the carotid artery. The reflected ultrasound echoes were acquired by the same piezoelectric transducer. Considering a circular geometry for the arterial section, strong echo patterns from this narrow beam width ultrasound transducer (3 dB half angle < 1.3°) was observed only when the beam passes normal to both the walls [1]. Therefore, desired diameter parameters were evaluated by acquiring strong and sharp echoes from both proximal and distal walls by orienting the probe along the arterial diameter chord. Fully automated, intelligent signal processing algorithm of our ARTSENS® (ARTerial Stiffness Evaluation for Non-invasive Screening – an image-free device for vascular stiffness evaluation) technology [1-4] was used to identify the echoes from the arterial walls, track the wall motion and measure the arterial dimensions (ΔD and D_D) from each cardiac cycle. For the current application, the basic ARTSENS hardware and measurement software was optimized and is discussed in the following sections.

We had previously presented the design of a Hall-effect and permanent magnet-based MPG sensor for blood pulse detection from the carotid site [5, 6]. Accuracy and repeatability of carotid local PWV measurement using the duality of such MPG sensors were also demonstrated by in-vivo studies in laboratory settings [5]. The same measurement principle and design concepts were followed to realize the proposed bi-modal arterial compliance probe. As shown in S1 Fig, each MPG sensor unit (size = 4.1 mm × 9.8 mm) consisted of a permanent magnet and a highly sensitive Hall-effect magnetic sensor IC. The disc-shaped (diameter = 3.175 mm) permanent magnet used in the sensor (D032A – Amazing Magnets LLC) produced an ambient field, which encompasses the arterial site under measurement. Instantaneous fluctuation in the encompassed field due to volumetric change in circulating blood was detected by the Hall-effect magnetic sensor (SS49E T3 – SEC Electronics Inc.), and it produced an output voltage proportional to the propagating blood pulse waveforms [5]. Two identical MPG sensors were mounted over a ring-shaped sensor PCB (outer diameter = 28 mm, inner diameter = 9 mm), and linearly arranged on either side of the ultrasound transducer (S1 Fig) without any cross-sensitivity between individual sensing elements. Such an integrated arrangement was suitable for simultaneous acquisition of dual blood pulse waveforms along with the ultrasound echo signals. Blood pulse waveforms obtained from the MPG sensors were processed in real-time using dedicated algorithms to assess local ΔT from each cardiac cycle. The center-to-center distance between the proximal and distal MPG sensors (23 mm; shown in S1 Fig) was used as the blood pulse propagation distance (ΔX = 23 mm) to evaluate local PWV.

Measurement system architecture

A portable device (S2A Fig) that operated in conjunction with a Microsoft® Windows® based tablet computer (Acer Aspire P3) was developed to validate the principle of cuffless, calibration-free evaluation of carotid ΔP using the arterial compliance probe. This prototype offered full control over simultaneous measurement of local PWV, ΔD, and D_D, as well as real-time ΔP evaluation from continuous cardiac cycles. A custom-designed graphical user interface (GUI) was used in the touchscreen tablet for easy operation. The device hardware (S2B Fig) includes all sections required to excite the transducer/sensing elements and continuously acquire the response and output physiological signals.

Electronics module for ultrasound transducer

An overall system architecture of electronics hardware of the prototype device has been presented in S3 Fig. The ultrasound hardware section included a high voltage transducer excitation circuit and high-speed analog signal acquisition circuit. A train of 4 pulse signals each with 100 ns pulse width was generated at a fixed time interval (F_S) using a microcontroller (ARM cortex-M4 – NXP semiconductor). These pulses were level transformed to a high voltage (±40 V) square wave pulse train by a high-speed ultrasound pulser IC (STHV748 – ST

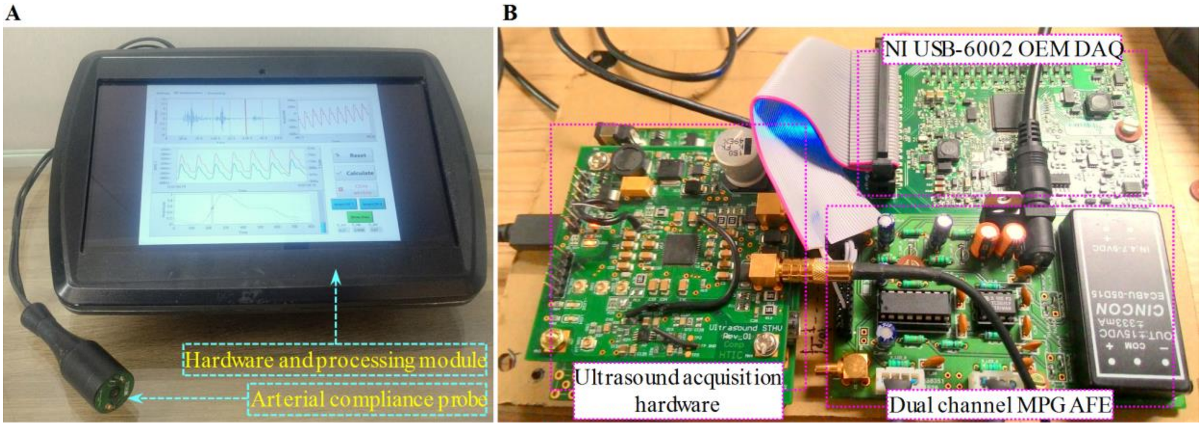

S2 Fig. Developed proof-of-concept prototype device for local ΔP measurement.
(A) Local ΔP device and arterial compliance probe used for experimental validation of the proposed non-invasive, cuffless technique. (B) Electronics hardware board used in local ΔP prototype device.

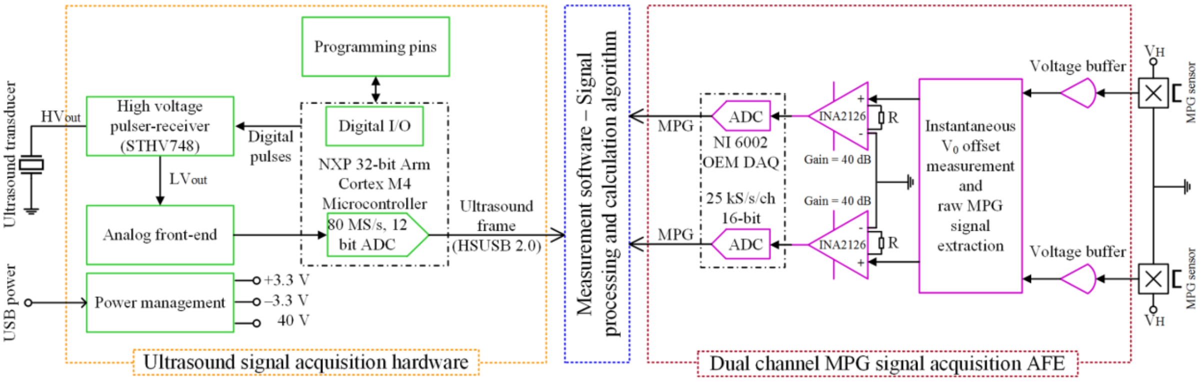

S3 Fig. Hardware architecture of the ultrasound and MPG signal acquisition and pre-processing units.

Microelectronics). These short duration high voltage square pulses were then used to excite the single-element ultrasound transducer in order to generate ultrasound pulse waves at its characteristic center frequency (5 MHz). After sending short duration ultrasound pulse waves towards the measurement site, the transducer was switched to receiving mode so as to capture the ultrasound echoes and convert into electrical signals. The received electrical signals were initially passed through low noise amplifier and active bandpass filters with a cutoff frequency of 1 MHz to 8 MHz. Dedicated onboard analog-to-digital converter (ADC; sampling rate = 80 MS/s, resolution = 12-bit) was used to digitize the pre-processed ultrasound echo signals. In the current hardware design, a single ultrasound echo frame consisted of echo signal acquired and digitized for approximately 50 – 65 μs. Considering 1540 m/s speed for the sound wave in human tissue, each echo frame has a depth of vision of around 40 – 50 mm. The time interval F_S between two pulse trains was kept at 12 ms resulting in 82 consecutive ultrasound echo frames per second, which was sent to the tablet computer via HSUSB 2.0 communication channel.

Analog front-end for MPG sensors

Electronics hardware of the local PWV module comprised of an application-specific analog front-end (AFE) circuit (S3 Fig) to simultaneously acquire dual blood pulse signals. The AFE unit had two independent channels for proximal and distal MPG signal acquisition. Hall-effect ICs of the arterial compliance probe was connected to the AFE using shielded cables and operated over single supply voltage (V_H = 5 V). The output voltage from the Hall-effect ICs was initially received by the voltage followers implemented in each channel. Raw MPG waveforms contain a pulsatile physiological signal (in units of mV) proportional to the arterial blood pulse propagation, which was superimposed on the quiescent offset voltage (V_0; in units of V) of the Hall-effect IC. Removal of this time varying offset voltage from the MPG signal before the amplification stage was critical to avoid the permanent saturation of amplifier IC. Specially designed subtractor circuits were used to remove the instantaneous V_0 voltage level in order to extract the pulsatile components from raw MPG signals. This subtractor-based approach (instead of using conventional signal conditioning filters with energy storage elements such as a capacitor) significantly reduced the error in local PWV due to the inherent inter-channel delay between the analog channels of AFE [5]. Pulsatile components of the MPG signals were further amplified (gain ≈ 40 dB) using a dual channel instrumentation amplifier (INA2126P – Texas Instruments). These MPG signals were digitized using National Instruments' NI USB-6002 OEM data acquisition module (DAQ; sampling rate = 25 kS/s/ch, resolution = 16-bit), and sent to the tablet computer via Hi-Speed USB cable.

Algorithms and signal processing

Ultrasound echo frames and MPG waveforms digitized from respective electronics hardware modules were synchronized with respect to system time before transferring to the tablet computer. These signals were successively used for real-time processing and analysis in the digital domain by means of application-specific algorithms. A custom virtual instrumentation program developed in National Instruments' LabVIEW platform (measurement software) was used for the signal interpretation, processing, pulse wave analysis, and evaluation of desired

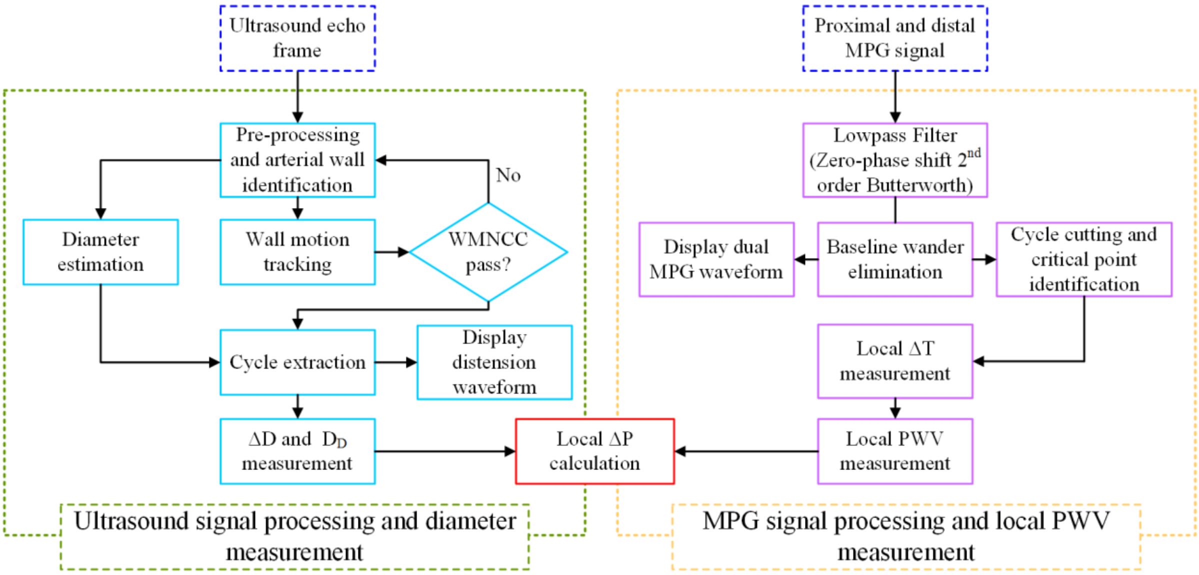

S4 Fig. System architecture of the developed signal processing and measurement software.

physiological parameters. Ultrasound data and dual channel MPG signals were processed simultaneously, but independently and the beat-by-beat measurements along with their repeatability indices was displayed.

Measurement of arterial dimensions

The acquired RF echo frames were processed through custom designed fully automated algorithms implemented in the measurement software. In absence of image feedback from ultrasound, it becomes extremely important to provide feedback to the operator for the placement of the ultrasound probe. A simple and intuitive GUI front panel was developed on LabVIEW's virtual instrumentation platform. We have demonstrated automated measurement algorithms [1-3, 7] and the signal quality evaluation algorithms [8] in a series of publications. In this section, the ultrasound echo signal processing and arterial diameter measurement algorithms are described in brief with relevant citations for full algorithms. Various stages of ultrasound signal processing and arterial diameter waveform extraction has been depicted in S4 Fig using a simplified flowchart.

The automated algorithms were developed to identify artery walls by a temporal motion of the arterial walls and its typical diameter range. In this technique, arterial walls were identified by checking opposite motion of proximal and distal arterial wall [9]. Once the approximate locations of arterial walls were identified by algorithms, front panel indicator indicated the operator to keep ultrasound probe in the same position on patient's neck for the next few cardiac cycles. A window of length about 2 mm around proximal and distal arterial wall echo was selected and the identified wall locations were continuously tracked for subsequent echo frames. Cross correlation-based technique (wall motion negative correlation check – WMNCC) was also applied in this selected section to calculate spatial shift during successive echo frames. This technique has been described in past publications by our group [1], A temporal change in the arterial diameter was determined by this procedure which provided a maximum change in arterial diameter (ΔD; in units of mm) from systole to diastole.

A mathematical model and curve-fitting based technique were developed to estimate lumen start and end points on echo frames in real time [7]. Each cardiac cycle's end-diastolic frames were passed through this algorithm for end-diastolic diameter (D_D; in units of mm) calculation. This process was done over continuous cardiac cycles and beat-by-beat ΔD and D_D values were estimated.

During the tracking phase, the signal quality of the acquired ultrasound echo signals was inspected continuously by an application-specific signal quality parameterization algorithm [8]. Measurements were performed only if the custom-defined ultrasound echo's signal quality index was found to be above the satisfactory limits. Otherwise, the system returned to arterial wall identification stage and indicated operator to reposition the arterial compliance probe in order to get good quality and strong arterial wall echoes.

Measurement of local PWV

The flowchart illustrated in S4 Fig shows the sequence of dual MPG pulse wave analysis in the digital domain. At first stage, the waveform from both proximal and distal sensors was filtered using a 2nd order zero phase-shift Butterworth low-pass filter (cutoff frequency = 10 Hz). Zero phase shift filters were preferred to overcome any phase difference between the raw and filtered signal. Further, a cycle cutting algorithm was applied to proximal and distal MPG waveforms with the same time-window in both the channels. The segregated cycle pairs corresponding to each heartbeat from the proximal and distal MPG sensor were collated with their absolute time stamp, and successively used to evaluate local ΔT in a beat-by-beat manner. A specific point in the blood pulse cycle corresponding to the maxima of its first derivative signal was considered as a unique identification point (critical point) to measure local ΔT. This approach is referred to as first derivative maximum technique [10]. It was realized by differentiating each cycle pair with respect to time, and the time delay between their respective critical points was recorded as local ΔT. Measured transit-time values were used in the fundamental distance-time equation (S4 Fig) to obtain beat-by-beat local PWV (in units of m/s).

Beat-by-beat local ΔP evaluation

The absolute local ΔP level at the carotid site was calculated (in units of mmHg) from each cardiac cycle using simultaneously measured local PWV, ΔD, and D_D by substitution into (4) in real-time. Erroneous estimation of local ΔP may occur during the real-time assessment, due to non-ideal values of local PWV, ΔD, or D_D caused by breathing artifacts, unstable probe to skin attachment and/or degraded signal quality. These non-ideal effects were not considered in the blood pulse wave analysis algorithm. Therefore, a real-time screening test was performed on measured beat-by-beat local PWV, ΔD and D_D values before the final calculation, in order to discard all non-ideal values and corresponding pulse cycles. The occurrence of non-ideal values was indicated in real-time – this assisted the operator to re-orient the arterial compliance probe to acquire waveforms with desired signal quality.

References

1. Joseph J, Jayashankar V. A virtual instrument for automated measurement of arterial compliance. J Med Devices. 2010 Dec 03;4(4):045004. doi:10.1115/1.4002493.
2. Joseph J, Radhakrishnan R, Kusmakar S, Thrivikraman AS, Sivaprakasam M. Technical validation of ARTSENS-an image free device for evaluation of vascular stiffness. IEEE J Transl Eng Health Med. 2015 May 12;3:1900213. doi: 10.1109/JTEHM.2015.2431471.
3. Sahani AK, Joseph J, Sivaprakasam M. Evaluation of the algorithm for automatic identification of the common carotid artery in ARTSENS. Physiol Meas. 2014 Jul;35(7):1299-317.
4. Joseph J, Shah MI, Sivaprakasam M. ARTSENS® Pen: a portable, image-free device for automated evaluation of vascular stiffness. In: Proceedings of the IEEE International Symposium on Medical Measurements and Applications (MeMeA); 2016 May 15-18; Benevento, Italy. IEEE; 2016. p. 1-6. doi: 10.1109/MeMeA.2016.7533787.
5. Nabeel PM, Joseph J, Sivaprakasam M. A magnetic plethysmograph probe for local pulse wave velocity measurement. IEEE Trans Biomed Circuits Syst. 2017 Oct;11(5):1065-76.
6. Nabeel PM, Joseph J, Sivaprakasam M. Arterial compliance probe for local blood pulse wave velocity measurement. In: Proceedings of the 37th Annual International Conference of the IEEE Engineering in Medicine and Biology Society (EMBC); 2015 Aug 25-29; Milano, Italy. IEEE; 2015. p. 5712-15. doi: 10.1109/EMBC.2015.7319689.
7. Sahani AK, Joseph J, Radhakrishnan R, Sivaprakasam M. Automatic measurement of end-diastolic arterial lumen diameter in ARTSENS. J Med Devices. 2015 Aug 06;9(4): 041002. doi: 10.1115/1.4030873.
8. Shah MI, Joseph J, Sivaprakasam M. Ultrasound signal quality parameterization for image-free evaluation of arterial stiffness. In: Proceedings of the 36th Annual International Conference of the IEEE Engineering in Medicine and Biology Society (EMBC); 2014 Aug 26-30; Chicago, IL, USA. IEEE; 2014. p. 2326-29. doi: 10.1109/EMBC.2014.6944086.
9. Sahani AK, Shah MI, Joseph J, Sivaprakasam M. An improved method for detection of carotid walls in ARTSENS. In: Proceedings of the 36th Annual International Conference of the IEEE Engineering in Medicine and Biology Society (EMBC); 2014 Aug 26-30; Chicago, IL, USA. IEEE; 2014. p. 1957-60. doi: 10.1109/EMBC.2014.6943996.
10. Runciman J, McGregor M, Silva G, Monteith G, Viel L, Arroyo LG. A new statistical phase offset technique for the calculation of in vivo pulse wave velocity. Artery Research. 2016 Mar;13:17-27.
